# Radiation-induced breast angiosarcoma: a case report

**DOI:** 10.3332/ecancer.2016.697

**Published:** 2016-11-29

**Authors:** Sara Tato-Varela, Rosa Albalat-Fernández, Sara Pabón-Fernández, Diego Núñez-García, Manolo La Calle-Marcos

**Affiliations:** 1 Clinical Management Unit of Gynaecology and Obstetrics, Hospital Universitario Virgen Macarena, Seville 41009, Spain; 2 Pathological Anatomy Service, Hospital Universitario Virgen Macarena, Seville 41009, Spain; 3 Family and Community Medicine, Hospital Universitario Virgen Macarena, Seville 41009, Spain

**Keywords:** breast cancer, radiation, secondary angiosarcoma, breast reconstruction

## Abstract

Radiation-induced breast angiosarcoma is a severe but rare late complication in the breast-preserving management of breast cancer through surgery and radiotherapy [1]. Often the initial diagnosis of this entity is complex given its relatively anodyne nature and usually being present in the form of typically multifocal reddish-purple papular skin lesions [2]. Because of the low incidence of this tumour, there is a limited number of studies regarding its optimal therapeutic management [3]. The preferred treatment is aggressive surgical removal and the prognosis is poor with an overall survival rate of 12–20% at five years [4].

## Introduction

In recent decades breast-preserving surgery with adjuvant radiotherapy has replaced mastectomy as the standard treatment of breast cancer in the initial stages [1]. Breast angiosarcomas following radiotherapy are a rare entity of which the incidence is estimated at 0.05% for all breast cancers [2], and its diagnostic criteria include a history of radiotherapy and a latency period of 5–10 years following radiotherapy [3]. The prognosis is poor with a high rate of relapse and an increased tendency to metastasise [4]. The following outlines a clinical case of radiation-induced breast angiosarcoma encountered in our centre as well as a review of the literature regarding this type of tumour.

## Clinical case

A woman of 62 years of age came to our centre’s Breast Unit in December 2014 following the appearance of skin lesions in the lower quadrants of her left breast. Her medical history included arterial hypertension, procedures in both upper limbs for osteomyelitis, and the 2006 treatment for a medullary carcinoma in the left breast, stage IA, by lumpectomy and axillary dissection (tumour of 0.8 cm grade III, RE 20%, RP negative, Ki67 57% and CerbB2 2+ and negative lymph nodes), having required adjuvant radiotherapy afterwards, which had been administered without incident.

A mammogram was performed which returned with a result of BIRADS 2, for which reason excision under local anaesthetic was conducted resulting in dermal angiosarcoma and changes attributable to radiotherapy. The tumour was 1 cm and occupied the reticular dermis with positive immunohistochemistry at CD31, CD34, absence of HHV8, and high rate of proliferation (80%).

In the first review, the patient again had multiple skin lesions in the inferior-internal and superior-external quadrants of the left breast. After having ruled out the existence of deep plane invasion by CT scan, it was decided to first remove the lesions under local anaesthetic. Having obtained the same result (dermal angiosarcoma not contacting the surgical borders), a bilateral mastectomy was subsequently performed after discussing the case with the Tumour Committee. The mastectomy and the postoperative period occurred without incident in May 2015. In the right breast no pathological findings were observed; in the left breast a focus of angiosarcoma was observed at the level of the deep dermis and subcutaneous tissue of 1 cm in diameter, probably radiation induced. The immunohistochemistry study was positive for CD31, CD34, FLI1 and there was evidence of four mitosis every 10 CGA as well as an increased Ki67.

In August 2015, the patient returned for consultation following the appearance of tumours in the left mastectomy scar. Five lesions (one in the sternal area and four in the pericicatricial area) were removed under local anaesthetic, again obtaining the result of angiosarcoma positive for CD31, CD34, ERG, and MYC which contacted the resection borders.

**Figure 1.**
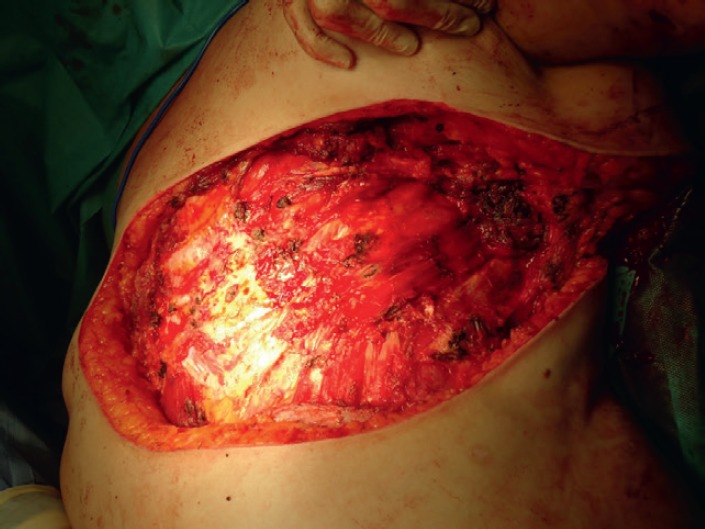
Radical excision of affected tissue including the pectoralis major muscle, fascia of the rectus, and fascia of the serratus anterior. The free edge of the latissimus dorsi is preserved.

**Figure 2.**
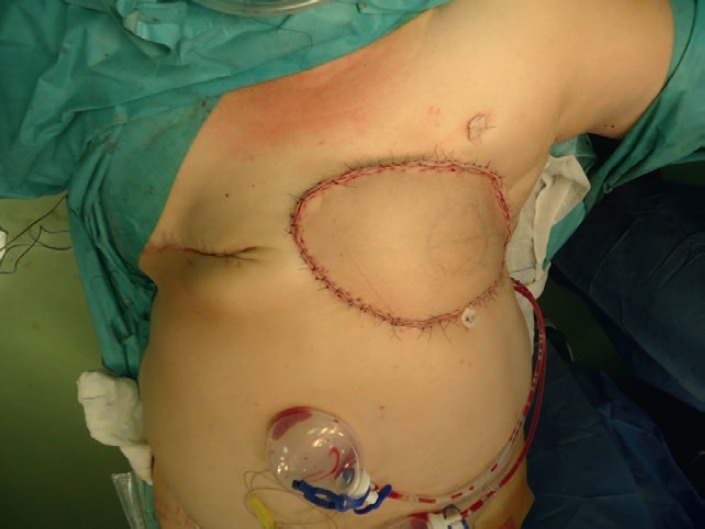
Result of the reconstruction with a free fasciocutaneous flap coming from the thigh.

In the Tumour Committee, it was decided to proceed with removal of all the affected skin together with plastic surgery and the completion of a flap to close off the affected skin. An MRI was performed which showed an absence of deep plane infiltration. In October 2015 a radical excision was performed including the pectoralis major muscle, fascia of the rectus, and fascia of the serratus anterior preserving the free edge of the latissimus dorsi (Figure 1). Reconstruction was subsequently performed using a free fasciocutaneous flap coming from the thigh (Figure 2). The postoperative period occurred without incident and the patient was discharged 14 days after the procedure and is asymptomatic up until the time of this writing.

## Discussion

One of the risks associated with the use of radiotherapy is the secondary development of malignant tumours. It is the cause for debate when compared to the breast-preserving management of breast cancer consisting of surgery and adjuvant radiotherapy [5]. Although the use of this type of treatment does increase the probability of developing a sarcoma in comparison to those patients who did not receive radiotherapy, the total risk is low and current evidence suggests that only 3–6% of all sarcomas are related to this adjuvant therapy [6]. It should be noted that the risk of developing secondary sarcoma is greater if the radiotherapy was performed at an early age or if the patient additionally received chemotherapy [7]. As early as 1983, Davies *et al* [8] reported the first case of mammary angiosarcoma after lumpectomy, lymphadenectomy, and radiation therapy for primary breast cancer.

Angiosarcoma is a highly aggressive vascular tumour characterised by a rapid proliferative pattern and a tendency to infiltrate any organ in the body. Mammary angiosarcomas may appear *de novo* (primary angiosarcomas) or as a complication of radiotherapy or chronic lymphoedema [9]. Classical or primary angiosarcomas usually affect the skin of the head and neck in elderly patients and are the most common variant (50–60%). Those associated with chronic lymphoedema appear after a variable period (4–27 years) almost always in the limb with chronic lymphadenopathy secondary to radical mastectomy with axillary lymphadenectomy (Stewart-Treves syndrome) [4]. Angiosarcomas secondary to radiotherapy meet Cahan's criteria: history of exposure to radiotherapy, histological discordance between primary and secondary tumour, and a long latency period between radiation therapy and the appearance of sarcoma [10].

Contrary to what occurs in primary angiosarcoma of the breast, which usually appears in women aged 30–50 years, radiation-induced angiosarcoma affects older women (with a median age of 67–71 years) about 10.5 years after radiotherapy as treatment of primary breast cancer (the median onset latency varies from 5–10 years). It has been proposed that with radiotherapy doses greater than 50 Gy, cellular apoptosis occurs; however, with doses below this level, DNA damage and genomic instability occur. Probably because of this, the secondary sarcomas appear at the edges of the radiation fields where the radiotherapeutic dose is more heterogeneous [11].

The pathogenesis of radiation-induced breast angiosarcoma is unclear. It is believed that radiation triggers both genomic instability and a mutation of relevant oncogenes. All molecular studies carried out on radiation-induced angiosarcomas present inactivation of the p53 gene as well as the expression and amplification of the MYC oncogene in the 8q24 region [12], although its absence does not exclude the diagnosis. Steth *et al* [13] speak in favour of an association between radiation-induced secondary angiosarcomas and mutations of the BRCA1 and BRCA2 genes because of increased radiosensitivity in these patients as well as increased susceptibility to carcinogenesis in the resulting cells.

Radiation-induced angiosarcoma usually affects the cutaneous dermis, although occasionally it may develop in the parenchyma. Initially, it may resemble a haematoma (flat violaceous lesions), i.e. appear in the form of reddish purple multifocal nodules or adopt the appearance of eczema [9]. When tumour size increases, ulcers or oedema may appear. Other symptoms such as pain are uncommon [2]. Growth is explosive in high grade tumours and more insidious in low grade tumours. Its differential diagnosis includes cutanides erysipelatoid carcinoma (cutaneous metastases similar to erysipelas) and atypical vascular lesions which appear 3–6 after radiation therapy for breast cancer [4].

When examined with ultrasound, these lesions present variable characteristics, being able to adopt a hypoechoic, hyperechoic, or heterogeneous appearance with or without posterior acoustic shadowing [14]. Radiographically, the finding of skin thickening by mammography or nuclear magnetic resonance (MRI), or the appearance of skin lesions that enhance the contrast in the MRI may raise the suspicion of this secondary tumour lesion [2].

**Figure 3.**
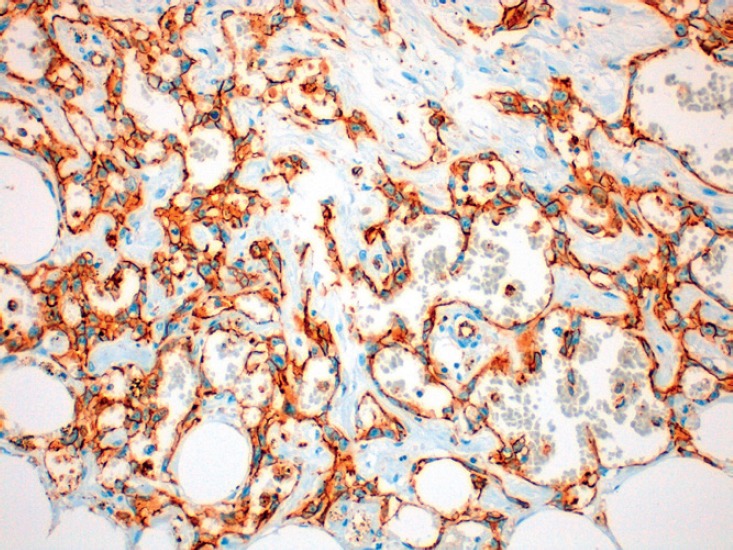
20x amplification of the definitive anatomopathological study of our case where the positivity of CD31 is observed.

The histological diagnosis of moderately or poorly differentiated radiation-induced angiosarcomas is relatively straightforward. It shows factor VIII and PECAM-1 (CD31) immunostaining positive malignant endothelial cells (Figure 3) which form vascular channels and extend into the surrounding stroma [15]. Well-differentiated angiosarcomas can be difficult to diagnose and have to be differentiated from hemangiomas and from atypical vascular lesions that appear after radiotherapy [16]. These latter lesions do not usually exhibit elevated mitosis or cellular atypia, although the cells may be hyperchromatic and form anastomotic vascular clusters with varying degrees of inflammation [15]. It is worth noting that in radiation-induced angiosarcomas, there is an increased v-myc myelocytomatosis viral oncogene homolog that does not exist in the atypical vascular lesions. This could be used to reach the diagnosis in complex cases or before limitation of available tissue [4].

The treatment of choice is aggressive surgery [4] with a view to the removal of the lesion with a safety margin. Seinen *et al* [1] report in their series that mastectomy is more likely to reach R0 (defined as free margin >2cm), although high recurrence rates are observed in the first six months (14 of 23 patients). Other authors suggest that the lower border is the most frequently affected in the case of incomplete resection for which reason aggressive resections involving the muscle may be considered in collaboration with plastic surgeons for subsequent reconstruction [11].

It has been postulated that the adjuvant use of hyperfractionated and accelerated radiotherapy could achieve a better control of the disease after surgery [4]. In the case of inoperable or advanced disease, treatment with chemotherapy is recommended. Angiosarcomas are particularly sensitive to taxanes and liposomal doxorubicin, so their weekly administration may be considered an alternative to traditional anthracycline treatment with ifosfamide. It should be noted that the associated toxic effects (cardiac and neurological) do not allow the prolongation of this therapy beyond 6–7 months usually. It is limited also because of existence of secondary resistance after response being frequent [9].

The prognosis of primary mammary angiosarcomas is directly related to histological grade and size [12]. Advanced stage diagnosis justifies the poor prognosis of most patients. Molecular studies of radiation-induced angiosarcoma specimens by genomic hybridisation suggest that gains in 7q or 8q are associated with a poorer prognosis [17]. Survival is generally poor, averaging around three years [14].

## Conclusion

Radiation-induced angiosarcomas of the breast are a rare and aggressive type of tumour which we must suspect before the appearance of erythematous nodules in a breast previously exposed to radiation therapy. Its treatment of choice is aggressive surgery, i.e. it involves removal of the pectoral muscle and needs subsequent reconstruction to achieve free margins. In advanced or inoperable cases, good responses to taxanes and liposomal doxorubicin chemotherapy have been described. The overall prognosis is poor, worsening in the case of poorly-differentiated tumours

## Conflicts of interest

The authors declare that there are no conflicts of interest.

## Contributions of the authors

All of the authors participated in the bibliography compilation. STV was responsible for the preparation of the manuscript. All authors approved the final version of the manuscript.
